# Practical Notes and Formulæ

**Published:** 1888-05

**Authors:** 


### PRACTICAL NOTES AND FORMULÆ.

**Salicylic Collodium.**—The following formula is recommended *Rundschau*, No. 873, 1887, as a most efficacious application for corns, warts, or any hardened flesh:

℞. Acid. salicylici.......................... 10 parts,
Acid. lactici.............................. 10 parts,
Collodii................................... 80 parts.

M. S.—Salicylic collodium.—*Therapeutic Gazette.*

**Insect Stings.**—Dr. Bernbeck, in the *Vereinsblatt der Pfalzer Aertze*, No. 6, advises the following applications for insect stings or bites:

1. ℞. Collod. elast......................... 19 (ʒv),

Acid. salicyl.......................... 1 (15½ gr.).

S.—To be applied to the sting.

2. ℞. Collod. elast......................... 10 (ʒ iijss),

Hydrarg. bichlorat. corrosiv............ .01 (1/6 gr.).

S.—As above.

Both of the above lotions are equally good, so that ammonia need no longer be used in such cases. As soon as the lotion is applied the pain and irritation ceases, and only rarely did the surrounding skin become swollen in consequence of the sting,—that is, when the remedy was immediately applied.—*Therapeutic Gazette.*

**Formulæ for Purgative Mixtures.**—In obstinate constipation:

℞. Ol. ricini................................ ʒ viiss,

Syrup. rhei.............................. ʒ v,

Alcohol.................................. ʒ iij¼,

Essent. menth. piper..................... gtt. ij.

In one dose or two as needed.—*Maryland Medical Journal.*

An agreeable powder or mass results from the combination of the following ingredients:

℞. Rad. jalap. pulv.......................... 1 part,
Sennæ fol. pulv.......................... 1 part,
Sacchar. albæ............................ 1 part,
Tamarind. pulp........................... 6 parts.

Which may be partially dried and made into chocolate-covered tablets.—*Revue de Therapeutique.*

**Try** the following prescription to abort an attack of acute bronchitis. Prof. H. C. Wood says that it is worth $5,000 to every medical student:

℞. Potassii citratis............................................℥ j,

Syrupi ipecacuanhæ........ ... ...............f℥ j,

Succi limonis............................... ......℥ ij,

Aquæ.................................................℥ iij,

M. S.—Two teaspoonfuls every three hours.—*Medical Times.*

**Agaricin in Phthisis.**—Agaricin is recommended by Jonng as a preventive of the weakening perspiration so frequently met with in phthisis He advises its use in the following formula:

℞. Agaricini.......................50 grm. (gr. vii 7-10),
Pulv. Doveri...................7.50 grm. (gr. cxvi),
Gummi arab....................
Pulv. altheæ....................aa 4 grm. (gr. lxi 7-10).

F. pil. ne. centum. S.—1 to 2 pills daily.

By the use of agaricin in the above form its laxative properties are reduced.—*Therapeutic Gazette.*

**For Inhalation in Phthisis.**—Filleau and Petit have found a most useful preparation of inhalation:

℞. Acid. carbolic.....................................gr. xxx,

Essent. terebinth...................................ʒ xij ss,

Essent. picis..................... ...............ʒ v,

Eucalyptol, (Merck)..........................ʒ vij ss,

Chloroform......................................gtt. v.

Inhalations of five minutes each are devised, four or six times daily.—*Revue de Therapeuteque.*

**A Laxative Gastric Tonic.**—Bardet has used to advantage:

℞ Ext. fluid. cascara sagrad.........................ʒ x,

Tinct. nucis vom.....................................*m* 30.

Aquæ destil............................................ʒ xxviij¾,

Syrup. simpl..................... ............ʒ iij¾,

M. Sig.—Dose, a teaspoonful.—*Journal de Medicine.*

**A Mouth Wash.**—Take resorcin, 2 drachms; vol. ext. eucalyptus, 1 drachm; aquam, ad 4 ounces; mix, rub up with magnesium carbonate, 2 drachms, and filter. One teaspoonful to the tumbler of water, used frequently as a wash for spongy gums, stomatitis, or after extraction, will be found valuable.—*British Dental Journal.*

**Pain Obtunder After Extraction.**—L. P. Bethel, D.D.S., in *Ohio Dental Journal of Dental Science:* Take sulphuric ether, 1 ounce; oil of cloves, 3 drops; and carbolic acid, 1 drop. Apply to the cavity on cotton, and let it remain a few minutes before removing.—*Medical Digest.*

